# Physical and Geometrical Properties of Additively Manufactured Pure Copper Samples Using a Green Laser Source

**DOI:** 10.3390/ma14133642

**Published:** 2021-06-29

**Authors:** Samira Gruber, Lukas Stepien, Elena López, Frank Brueckner, Christoph Leyens

**Affiliations:** 1 Fraunhofer Institute for Material and Beam Technology, IWS, Winterbergstraße 28, 01277 Dresden, Germany; Lukas.Stepien@iws.fraunhofer.de (L.S.); elena.lopez@iws.fraunhofer.de (E.L.); Frank.Brueckner@iws.fraunhofer.de (F.B.); christoph.leyens@iws.fraunhofer.de (C.L.); 2 Department of Engineering Sciences and Mathematics, Luleå University of Technology, 97187 Luleå, Sweden; 3 Institute of Materials Science, Technische Universität Dresden, Helmholtzstr. 7, 01069 Dresden, Germany

**Keywords:** additive manufacturing, laser powder bed fusion, pure copper, short wavelength laser system, green laser, eddy-current method, electrical conductivity

## Abstract

So far, copper has been difficult to process via laser powder bed fusion due to low absorption with the frequently used laser systems in the infrared wavelength range. However, green laser systems have emerged recently and offer new opportunities in processing highly reflective materials like pure copper through higher absorptivity. In this study, pure copper powders from two suppliers were tested using the same machine parameter sets to investigate the influence of the powder properties on the material properties such as density, microstructure, and electrical conductivity. Samples of different wall thicknesses were investigated with the eddy-current method to analyze the influence of the sample thickness and surface quality on the measured electrical conductivity. The mechanical properties in three building directions were investigated and the geometrical accuracy of selected geometrical features was analyzed using a benchmark geometry. It could be shown that the generated parts have a relative density of above 99.95% and an electrical conductivity as high as 100% International Annealed Copper Standard (IACS) for both powders could be achieved. Furthermore, the negative influence of a rough surface on the measured eddy-current method was confirmed.

## 1. Introduction

Copper has the second highest thermal and electrical conductivity of all non-superconducting materials, making it the material of choice for many functional applications such as heat exchangers or induction coils. Additive manufacturing processes offer a high degree of geometrical freedom for the fabrication of complex parts and are therefore a promising technology for pure copper applications. So far, pure copper has been processed via electron beam melting (EBM) [1,2,3,4], binder jetting (BJ) [5], laser powder bed fusion (LPBF) [6,7,8,9,10,11,12,13,14] and laser metal deposition (LMD) [15,16,17]. The highest relative densities of 99.95% and electrical conductivities of 96.24% IACS up until 2020 were achieved with the EBM process [2,3,4]. Bai and Williams [5] reached 97.3% relative density after sintering with the BJ process. Due to the high thermal conductivity of pure copper and low absorptivity in the range of 25% [11,18] of the copper powder at laser wavelengths of 1000–1100 nm, which are mostly used for LPBF [19], stable processing is not possible for infrared laser powers below 500 W and, therefore, fully dense parts could not be produced [13,20]. So far, only relative densities of 83–88% have been reported for pure copper parts when using a common 200 W infrared laser source [14,21]. One approach to increase the relative density of additively manufactured pure copper parts is by increasing the laser power. Colopi et al. and Ikeshoji et al. [12,22] have reached relative densities as high as 99.1% to 99.6% when using laser powers of up to 1 kW at a wavelength of 1 µm. However, melt-pool instabilities still occur mainly due to the different absorptivity of the powder bed and the molten copper at those wavelengths, leaving parts with low surface quality. By far the most promising approach is using a laser source with shorter wavelengths, primarily “green lasers” with wavelengths of about 515 nm. Such a setup can produce additively manufactured pure copper parts with relative densities of up to 99.8% and an electrical conductivity reaching up to 100% IACS [23], thus enabling the highest performances. A green laser source was already used for LMD [16] and even blue diode lasers showed promising results in dense single tracks [17].

The aim of this study was to verify the robustness of the process by using two powders with different particle size distributions and to take a closer look at the evolving density, microstructure, and effect of geometry features such as wall thickness and surface condition on electrical conductivity measurements. The geometrical accuracy was analyzed via 3D scanning and the mechanical properties were also determined for three building directions.

## 2. Experimental Setup

### 2.1. Powder

Pure copper powders from two different powder suppliers were used: deoxygenated oxygen-free pure copper (Cu-OF) and oxygenated electrolytic tough pitch copper (Cu-ETP). Both powders were gas atomized. The particle size distribution and morphology were verified using the CAMSIZER X2 (Microtrac Retsch GmbH, Haan, Germany) based on the dynamic digital image analysis according to ISO 13322-2. The chemical composition was provided by the material suppliers and hot carrier gas extraction using the inductar^®^ ONH cube (Elementar Analysensysteme GmbH, Langenselbold, Germany). The absorptivity of the powders was measured using a UV-VIS Zeiss MCS400 (Carl Zeiss Spectroscopy GmbH, Jena, Germany) and FT-NIR spectrometer Bruker Vertex 70 (Bruker Corporation, Billerica, MA, USA). To verify the morphology and satellites, additional SEM imaging was used.

### 2.2. Machine

The samples were manufactured using a TruPrint 1000 Green Edition (TRUMPF GmbH + Co. KG (Holding), Ditzingen, Germany) with an integrated TruDisk1020 disk laser with a wavelength of 515 nm and a maximum laser power of 500 W. All specimens were processed with the same parameter set: line energy input 0.808 J/mm, hatch distance 120 µm, and a layer thickness of 30 µm.

### 2.3. Samples

**Figure 1:**
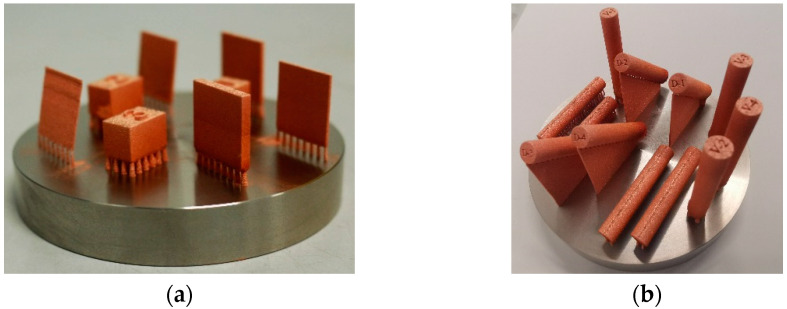
Steel build plate 100 mm in diameter with (**a**) density cubes 10 × 15 × 10 mm³ and vertical walls 20 × 20 mm² of different thicknesses and (**b**) cylinders with 8 mm diameter × 43 mm height for tensile samples in three building directions.

Copper cubes with 10 × 10 × 15 mm³ and vertical walls of 20 × 20 mm^2^ with different thicknesses of 500 µm, 1 mm, 1.5 mm, and 3 mm were built. Cylinders 8 mm diameter × 43 mm height for static mechanical testing were built in the vertical, horizontal, and 45° building direction (see Figure 1) and then machined to the geometry DIN 50125 B 4 × 20.

**Figure 2:**
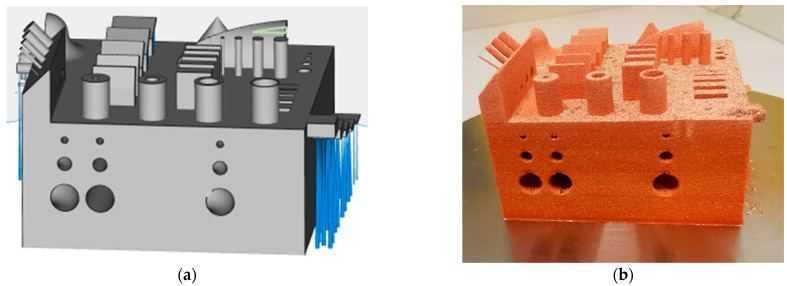
Benchmark geometry (**a**) build job preparation file with support structures (blue) and (**b**) pure copper structure built with Cu-OFHC.

To analyze the dimensional accuracy for certain features such as cylinders, overhangs, and walls, a benchmark geometry developed by the Fraunhofer Institute for Material and Beam Technology IWS within the AGENT-3D program was built and analyzed via 3D scanning using an ATOS Core GOM 135 (GOM GmbH, Braunschweig, Germany) which can be seen in Figure 2. The geometry and previous measurements with 3D scanning and computed tomography (CT) were described in detail by Lopez et al. [24] and Gruber et al. [25].

### 2.4. Sample Preparation

**Figure 3:**
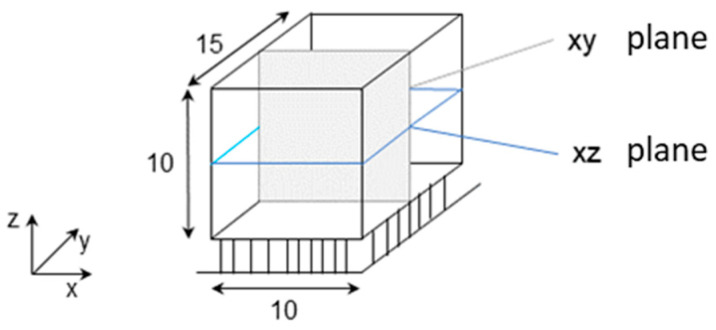
Definition of microsectioning planes.

After the LPBF process, the samples were separated from the steel substrate via electrical discharge machining. Each cube was microsectioned in two planes (x–y and x–z, see Figure 3) and prepared for optical porosity analysis via image analysis. The resulting porosity and mean pore sizes were averaged from a total of six microsections per powder and etched with Adler 10:1 for the analysis of the microstructure. 

The tensile cylinders were machined to B4x20 according to DIN EN ISO 50125 and tested with an inspect table 50 kN (Hegewald & Peschke Meß- und Prüftechnik GmbH, Nossen, Germany) according to DIN EN ISO 6892-1.

### 2.5. Conductivity Measurement

To measure the electrical conductivity, the eddy-current-based device SigmaScope 350 (Helmut Fischer GmbH, Sindelfingen, Germany) was used with an examination frequency of 120 kHz for the microsectioned cubes (x–z plane) and vertical walls (x–y plane) under the two surface conditions sand-blasted and milled. According to ASTM E1004—17 [26], the sample thickness should be at least 2.6 *δ* where *δ* is the standard depth of penetration of the induced eddy-currents calculated by Equation (1)

(1) $$\delta =\frac{660}{\sqrt{f\times \sigma }}\left(\mathrm{mm}\right)$$

where

*f* = examination frequency in Hz, dependent on the sensor, 

*σ* = electrical conductivity of the sample in IACS percentage

Each measurement was repeated six times on the same sample and averaged. The precision of the measurement was 0.09 MS/m for 120 kHz and the correctness less than 1% of the measured value according to Helmut Fischer GmbH. With an expected 100% IACS and examination frequency of 120 kHz, the standard depth of penetration was 190 µm. Following the rule of a minimum wall thickness of 2.6 δ required a minimum sample thickness of 500 µm.

## 3. Results and Discussion

### 3.1. Powder Analysis

#### 3.1.1. Morphology

**Figure 4:**
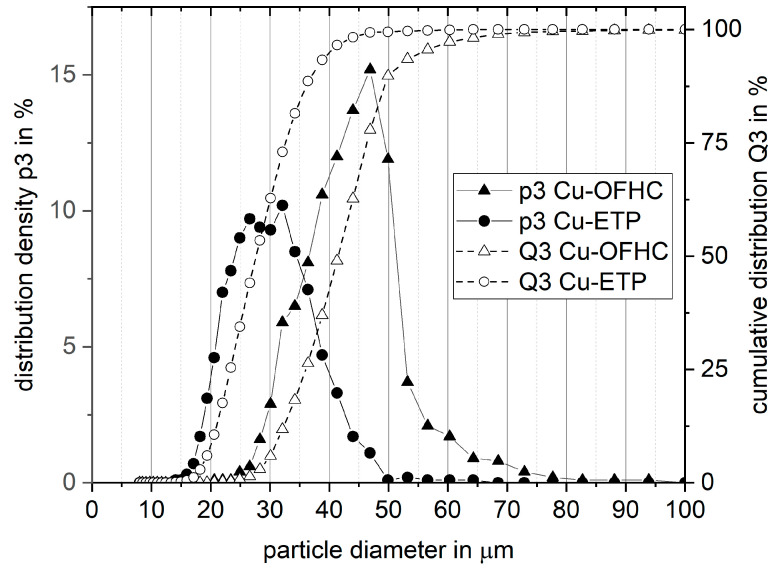
Particle size distribution of Cu-OFHC and Cu-ETP measured with the CAMSIZER X2.

The particle size distribution of both powders differed greatly as can be seen in Figure 4 and Table 1. D10 of Cu-OFHC was almost equal to D90 of Cu-ETP. Cu-ETP showed a bimodal behavior with peaks at 26 µm and 32 µm, whereas Cu-OFHC had a single peak at around 47 µm. 

**Figure 5:**
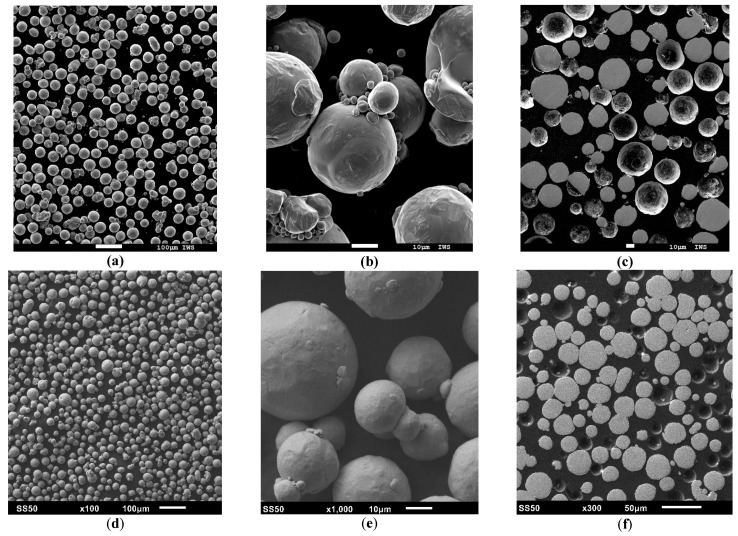
SEM images of loose Cu-OFHC powder at (**a**) 100× magnification, (**b**) 1000× magnification, and (**c**) embedded 300× magnification; SEM images of loose Cu-ETP powder at (**d**) 100× magnification, (**e**) 1000× magnification, and (**f**) embedded 300× magnification.

SEM imaging of both powders confirmed the presence of larger pores (see Figure 5) and the high sphericity values from the dynamic imaging showed a similar spherical shape and low amount of agglomerated particles. Additionally, no internal pores were observed for both powders from the embedded microsections.

#### 3.1.2. Chemical Composition

Despite the different grades provided, both powders had a similar chemical composition according to supplier specifications and hot carrier gas extraction measurements (see Table 2). One explanation for this could be the oxygen uptake of the Cu-OFHC powder during powder production and powder handling prior to processing, leading to similar results compared to the Cu-ETP powder. 

#### 3.1.3. Absorptivity

The absorptivity A of both pure copper powders at 515 and 1064 nm shown in Table 3 emphasize the advantages of using a green laser source, since the absorption increases by 260% compared to using a conventional infrared laser source.

Cu-ETP showed higher absorption for both wavelengths, which could be explained by the smaller particle size resulting in larger surface area and increased multiple scattering of the laser beam in the powder bed that was also observed by Gu et al. [27]. Overall, both powders had similar morphological and chemical properties and the main difference was the particle size distribution.

### 3.2. Porosity

**Figure 6:**
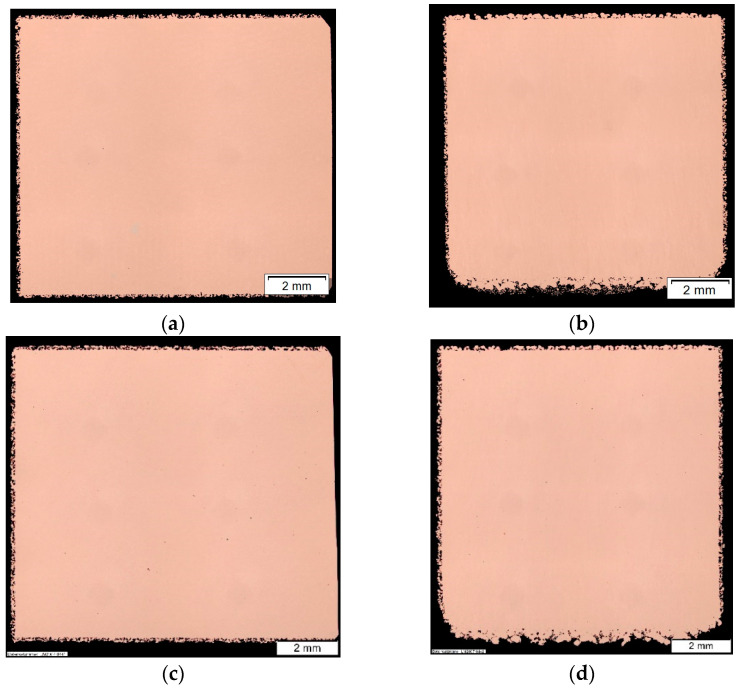
Polished microsection of Cu-OFHC in the (**a**) x–y plane and (**b**) x–z plane and polished microsection of Cu-ETP in the (**c**) x–y plane and (**d**) x–z plane.

Light microscopy of the two microsectioned planes of the density cubes revealed a dense core and porous surface for both powders (see Figure 6) with a core porosity of 0.013% for the Cu-ETP powder and 0.017% for the Cu-OFHC powder (see Table 4).

Again, the Cu-ETP exhibited slightly higher density values, which could be attributed to the smaller particle size resulting in higher packing and higher absorption. At the same time, it should be noted that microsections only reveal the behavior in one plane and the measured difference in density could also be statistically insignificant.

### 3.3. Microstructure

**Figure 7:**
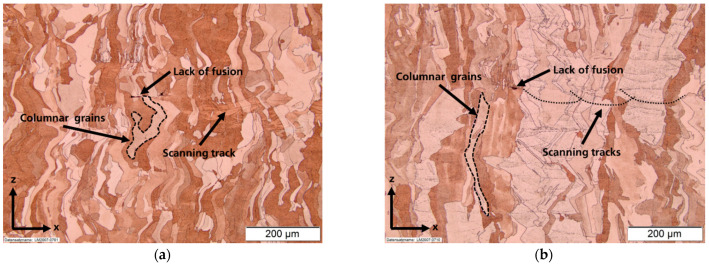
Microstructure of (**a**) Cu-OFHC in the x–z plane and (**b**) Cu-ETP in the x–z plane.

The microstructure in the x–z plane for both powders showed the typical grain growth parallel to the building direction (see Figure 7). Even though the measured porosity was very low, single spots of lack of fusion could be detected in both samples. The scanning tracks were also faintly visible and gave insight on the track width and penetration depth of the laser. The minimum track width was mainly restricted by the used 200 µm laser spot size. Density cubes from both powders showed similar microstructural evolution. 

### 3.4. Electrical Conductivity

The electrical conductivity was measured from the prepared x–z microsections of the previously analyzed three density cubes for each powder (see Table 5). 

**Figure 8:**
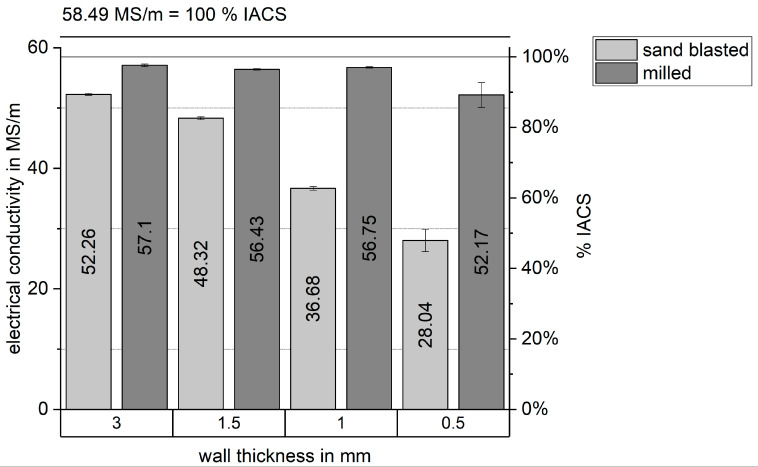
Effect of surface condition and wall thickness on measured electrical conductivity of Cu-OFHC.

The slight difference of measured electrical conductivity using three cubes built from both powders (0.7 MS/m or 1.4% IACS) could be insignificant. This should be investigated in a later study with more samples. The electrical conductivity measurements were continued for Cu-OFHC vertical walls of different wall thicknesses only, since the density measurements, microstructure, and bulk electrical conductivity for both powders were very similar. A large discrepancy of the electrical conductivity was observed between a sand-blasted surface and a milled surface (see Figure 8) and the maximum values from the bulk conductivity could not be reproduced. This effect of surface condition increased with smaller wall thicknesses. At a 3 mm wall thickness, the difference was 8.4% and at 1 mm wall thickness the discrepancy increased to 34.6% while the standard deviation also increased. It is thought that perhaps the remaining air in the porous layer (see Figure 6) acted as an isolator, which then reduced the measured conductivity. This could explain the lower conductivity measurements of the 500 µm wall. 

### 3.5. Static Mechanical Properties

The mechanical properties (see Table 6) were obtained only from the Cu-OFHC powder due to the low differences in density, microstructure, and electrical conductivity of the cubes from both powders. Significant anisotropic behavior was found in the three building directions. Vertically and horizontally built samples showed similar behavior, whereas the diagonal samples showed a lower Young’s modulus and lower strength. The mechanical properties of the additively manufactured copper were in the range of conventional soft annealed pure copper, according to [28] (see Table 6). 

### 3.6. Geometrical Accuracy

**Figure 9:**
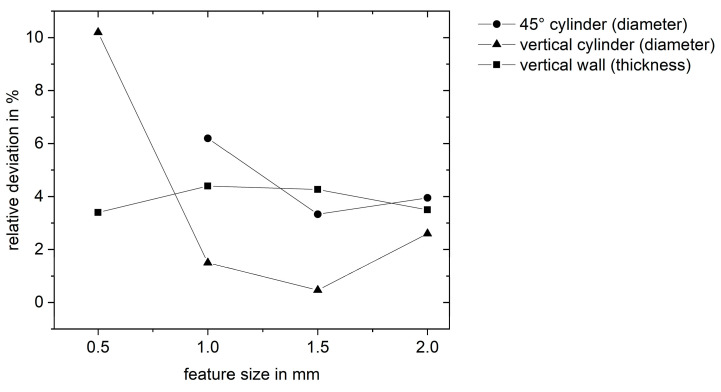
Relative deviation from the feature size of three different geometrical features (vertical wall, vertical cylinder, and 45° cylinder) analyzed from 3D scanning data points from the Cu-OFHC benchmark geometry.

Features smaller than 0.5 mm are not converted into scanning vectors in the slicing software Materialise Trumpf Build Processor and Magics 24.1 due to the set-up beam compensation of 100 µm in combination with the beam diameter of 200 µm. Therefore, the smallest features of the benchmark geometry with thicknesses of 100 µm were not scanned and could therefore not be analyzed. The geometrical accuracy was highest for vertical cylinders larger or equal to 1 mm in diameter (see Figure 9). The increased relative deviation at a diameter of 500 µm is attributed to the large laser focus and beam compensation. For vertical walls, the deviation was feature size independent around 3 to 4%. Overall, features larger than 500 µm had a very high accuracy. 

All cooling channels 1, 2, and 4 mm in diameter (straight and curved) could be built without support structures and the remaining powder could be removed with pressurized gas (see Figure 2b). This showed the potential for complex inner cooling channels in future pure copper applications using LPBF having an advantage compared to electron beam melting, where unused powder is agglomerated in a sinter cake, making the removal of powder in inner channels difficult to almost impossible.

## 4. Conclusions

The following conclusions could be made in this study:

- Density above 99.8% and bulk electrical conductivity values of 98.6% IACS and 100% IACS for the two pure copper powders with different particle size distributions were achieved in accordance with [23], proving that the integration of the green laser source into the TruPrint1000 results in a stable process to build high-quality pure copper samples.
- A ground or polished surface with an area of 15 × 15 mm^²^ and a sample thickness of at least 1.5 mm results in a reproducible and correct measurement of the electrical conductivity with the eddy-current method.
- The mechanical properties show anisotropic behavior, which was expected due to the layer-wise build up. The highest strength was found in horizontally build tensile samples.
- Features smaller than 500 µm are difficult to achieve due to the laser focus diameter of 200 µm, the scanning strategy of using contour lines for every feature, and the processing software.

Future work will focus on the influence of oxygen pick up within the powder during the build process and during powder storage on the part quality and powder recyclability as well as geometrical capabilities with the 200 µm laser focus diameter and minimum powder requirements for high-density and high-conductivity parts. 

## Figures and Tables

**Table 1:** Main characteristics of particle size distribution of Cu-OFHC and Cu-ETP.

Powder	D10 in µm	D50 in µm	D90 in µm	Sphericity
Cu-OFHC	31.6	41.5	50.1	0.919
Cu-ETP	19.5	26.2	34.9	0.923

**Table 2:** Cu content provided by the suppliers and H, N, O content of Cu-OFHC and Cu-ETP powder measured with hot carrier gas extraction.

	Supplier Information	Hot Gas Extraction			Technical Specification DIN CEN/TS 13388
	Cu	H	N	O	O
	in wt. %	in ppm			in %
Cu-OFHC	99.95	10	100	230	^1^
Cu-ETP	99.97	10	30	270	0.04–0.06

^1^ Oxygen content must ensure hydrogen resistance according to DIN EN 1976.

**Table 3:** Absorptivity A at 515 nm and 1064 nm measured with UV-VIS spectrometry.

Powder	A at 515 nm	A at 1064 nm
Cu-OFHC	72.21%	27.27%
Cu-ETP	76.93%	31.99%

**Table 4:** Porosity analysis of three density cubes per powder via image analysis.

Powder	Mean Porosity in %	Mean Max. Pore Size in µm	Mean Porous Layer Thickness in µm
Cu-OFHC	0.017 ± 0.0243	410.0 ± 8.50	152 ± 17.1
Cu-ETP	0.013 ± 0.0094	52.5 ± 27.59	144 ± 35.5

**Table 5:** Comparison of electrical conductivity measured with the eddy-current method on polished microsectioned surfaces.

	MS/m	% IACS
Cu-OFHC	58.12 ± 0.26	100.0 ± 0.44
Cu-ETP	57.34 ± 0.26	98.6 ± 0.44

**Table 6:** Tensile testing results of Cu-OFHC.

Condition (Building Direction or Conventional)	Young’s Modulus E in GPa	Yield Strength R_p0.2_ in MPa	Ultimate Tensile Strength R_m_ in MPa	Elongation at Break A in %
Vertical	130.6 ± 27.6	135.7 ± 2.3	212.3 ± 3.8	51.5 ± 8.4
Diagonal	90.0 ± 9.6	127.3 ± 2.1	187.7 ± 2.1	47.0 ± 3.2
Horizontal	144.3 ± 15	134.8 ± 2.5	224.3 ± 2.2	47.4 ± 3.5
Soft annealed Cu-OF acc. to [28]	110	<100	200–250	40–60

## Data Availability

The data can be requested from the corresponding author.
